# Applicability of a novel mathematical model for the prediction of adult height and age at menarche in girls with idiopathic central precocious puberty

**DOI:** 10.6061/clinics/2018/e480

**Published:** 2018-07-23

**Authors:** Mateus Cavarzan Lopes, Carolina Oliveira Ramos, Ana Claudia Latronico, Berenice B. Mendonça, Vinicius N. Brito

**Affiliations:** I Iniciacao Cientifica, Faculdade de Medicina FMUSP, Universidade de Sao Paulo, Sao Paulo, SP, BR; II Unidade de Endocrinologia do Desenvolvimento e Laboratorio de Hormonios e Genetica Molecular LIM/42, Disciplina de Endocrinologia e Metabologia, Faculdade de Medicina FMUSP, Universidade de Sao Paulo, Sao Paulo, SP, BR

**Keywords:** Adult Height, Central Precocious Puberty, Bayley-Pinneau Tables, GnRH analog, Age at Menarche

## Abstract

**OBJECTIVES:**

Unfavorable predicted adult height and psychosocial inadequacy represent parameters used to guide therapeutic intervention in girls with central precocious puberty. Gonadotropin-releasing hormone analog is the first-line treatment. The aim of this study was to compare two methods used to predict adult height and assess a validated tool for predicting the age at menarche in girls with central precocious puberty.

**METHODS:**

The predicted adult height of 48 girls with central precocious puberty was calculated at diagnosis using the Bayley-Pinneau method based on average and advanced bone age tables and compared with the predicted adult height calculated using a mathematical model. In addition, the age at spontaneous menarche was predicted using the new formulae. After Gonadotropin-releasing hormone analog treatment, the predicted adult height was calculated using only the Bayley-Pinneau tables.

**RESULTS:**

The achieved adult height was within the target height range in all treated girls with central precocious puberty. At diagnosis, the predicted adult height using the Bayley-Pinneau tables was lower than that using the mathematical model. After the Gonadotropin-releasing hormone analog treatment, the predicted adult height using the Bayley-Pinneau method with the average bone age tables was the closest to the achieved adult height. Using the formulae, the predicted age at spontaneous menarche was 10.1±0.5 yr. The Gonadotropin-releasing hormone analog treatment significantly postponed this event until 11.9±0.7 yr in these “idiopathic” central precocious puberty girls, highlighting the beneficial effect of this treatment.

**CONCLUSION:**

Both initial adult height prediction methods are limited and must be used with caution. The prediction of the age at spontaneous menarche represents an innovative tool that can help in clinical decisions regarding pubertal suppression.

## INTRODUCTION

Puberty that occurs in girls younger than 8 years is considered precocious (1,2). Precocious puberty is classified as central precocious puberty (CPP) if it occurs as a result of the premature activation of the hypothalamic-pituitary-gonadal axis. The term “idiopathic” (ICPP) is reserved for cases without central nervous system lesions and/or genetic defects 1,2. Long-acting GnRH analog (GnRHa) represents the treatment of choice for CPP 3. The aims of treatment include to preserve the genetic potential height and psychosocial adequacy 1-3.

The current methods used to predict adult height (AH) are highly inaccurate with a large margin of error 4-8. The most commonly used method is based on Bayley-Pinneau (BP) tables that predict AH based on bone age (BA). This method was developed for healthy children and can be highly inaccurate in CPP children who frequently have advanced BA 4-8. Recently, by performing a multiple linear regression analysis, Giabicani et al. 9 proposed new formulae from a mathematical model for height prediction in CPP girls. This model is based on the height, chronological age, parents' height and LH/FSH peak ratio after a GnRH stimulation test. These formulae were applied to both treated and untreated girls and were revealed to be more accurate in AH prediction than using BP tables at the time of CPP diagnosis 9. The main criteria indicating treatment in that cohort was a rapidly progressive form of CPP rather than a lower predicted AH. Consistently, the authors proposed a unique formula for predicting age at menarche that could be used as another clinical parameter to guide clinical decisions regarding whether to treat girls with CPP 9.

In this study, we tested this novel mathematical method for the prediction of AH and age at menarche in a cohort of patients with ICPP who were treated with GnRHa and achieved their final adult height and compared this method with the BP method in AH prediction.

## PATIENTS AND METHODS

This retrospective single-center study included 48 ICPP girls who were diagnosed and treated at the Unidade de Endocrinologia do Desenvolvimento do Hospital das Clínicas da Faculdade de Medicina da Universidade de São Paulo (FMUSP). The study design was approved by the local research ethics committee, and written informed consent was obtained from the patients and their parents.

The inclusion criteria are listed in Table 1. In total, 48 patients who were treated exclusively with GnRHa with an adequate clinical and hormonal suppression of pubertal development and reached their adult height were included in this study. The medical records of all patients were systematically reviewed for clinical, anthropometric and hormonal parameters at the time of CPP diagnosis, after the treatment, and after achieving adult height.

The hormonal profile (LH, FSH, and estradiol) was measured by performing ultrasensitive assays, including an immunofluorometric assay (IFMA) and electrochemiluminescence methods (ECLIA). The intra- and inter-assay coefficients of variation were < 5% in all assays. The thresholds used for diagnosis are shown along with the inclusion criteria in Table 1 1,2.

The BA was determined using the Greulich-Pyle atlas, which is a reference based on X-ray images obtained from the left hand and wrist from birth up to 18 years of age in girls and 19 years of age in boys 10,11. Ossification appears in the bones of the hand and wrist in a progressive and sequential order. Then, the BA is estimated by comparing the degree of ossification in the wrist and hand bones with the closest matching plate available on the atlas.

The predicted AH was calculated using the Bayley-Pinneau tables for advanced and average BAs 12,13. This method was developed using the percentage of the final height that a child has already reached based on his or her current BA 12,13.

**Figure 1:**
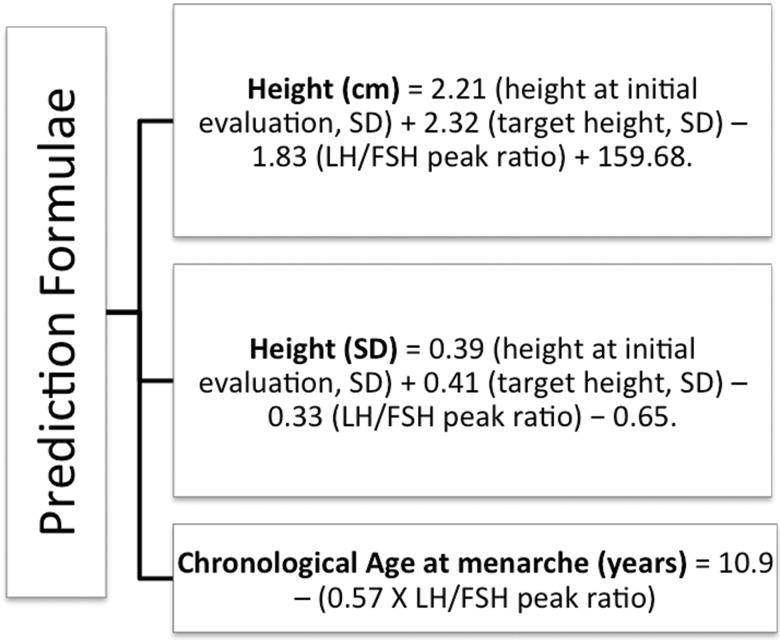
Formulae based on a mathematical model for the prediction of adult height and age at menarche.

The formulae from the mathematical model developed by Giabicani et al. 9 are shown in Figure 1 and are available online at http://www.kamick.org/lemaire/med/girls-cpp15.html.

### Statistical analysis

The data distribution was evaluated by performing a Kolmogorov-Smirnov test and was considered normal at *p*>0.05. The numerical data are presented as the means and standard deviation scores (SDS). The means were compared by performing Student's t-test, and the correlations between the variables were determined using the Pearson's correlation coefficient. The statistical software SigmaStat 3.5 for Windows (Systat Software, Inc.) was used for the analysis. Statistically significant results were set at *p*<0.05.

## RESULTS

### Initial clinical and hormonal data

**Figure 2:**
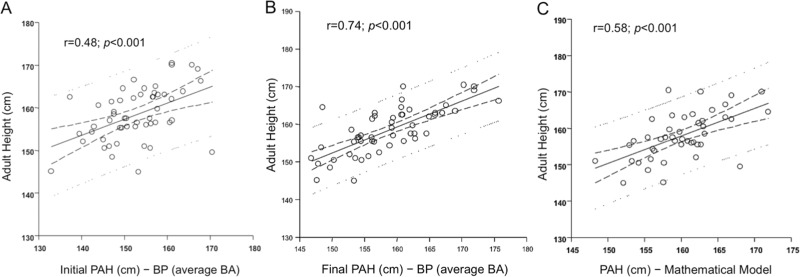
Correlations between adult height and predicted height using Bayley-Pinneau tables of average bone age at the time of diagnosis and (A) after treatment (B) and predicted height using the formulae from a mathematical model (C).

The clinical and hormonal data of 48 girls with ICPP are summarized in Table 2. The onset of pubertal signs occurred at a mean chronological age (CA) of 5.75±2.1 yr in the patients with ICPP. During the first medical evaluation, the mean CA was 7.75±1.7 yr, while the mean interval (Δ CA first evaluation – CA onset pubertal signs) to the diagnosis of CPP was 2±1.7 yr. At the onset of treatment with GnRHa, the mean CA was 7.9±1.2 yr, and the mean initial BA was 10.5±1.4 yr. At the time of the CPP diagnosis, the mean height was 134.2±8.9 cm. Regarding the puberty staging, according to Tanner's criteria for breast development (B), 5.7% of the patients were at stage B2, 41.5% of the patients were at B3, 50.9% of the patients were at B4, and 1.8% of the patients were at B5. The mean basal hormone levels were as follows: LH 1.6±1.3 U/L and FSH 4.0±1.85 U/L. After the GnRH test, the mean peak of LH was 17.5±15.5 U/L and FSH was 13.2±7.1 U/L. The mean LH/FSH peak ratio was 1.33±0.79. The initial PAH using the BP tables for average BA was 152.8±7.9 cm, while the initial PAH using the BP tables for advanced BA was significantly higher (156.5±8.6 cm) (*p*<0.05). Using the formulae from the mathematical model, the mean PAH at the time of the CPP diagnosis was 160±4.95 cm (mean SDS -0.6±0.88). At diagnosis, the positive correlations between AH and PAH using the BP tables for average or advanced BA (r=0.45 and r=0.48; *p*<0.001) were weaker than the correlations between AH and PAH using the formulae from the mathematical model (r=0.58; *p*<0.001) (Table 3 and Figure 2).

### Clinical data after the GnRHa treatment

The mean duration of the GnRHa treatment was 3.02±1.3 yr. After the GnRHa treatment, the mean CA was 10.9±0.7 yr, the mean BA was 12.5±0.7 yr and the mean height was 149.2±5.05 cm (SDS: 1.1±1.1).

After the GnRHa treatment, the mean final PAH was 158.8±6.7 cm using the BP method with the average BA tables and 160±7.15 with the advanced BA tables. Using the BP method, the mean PAH after the GnRHa treatment was significantly higher than the PAH at the start of treatment (*p*<0.001). The mean achieved AH was 158.4 ± 6.2 cm (SDS -0.63±1). The mean TH was 157.8±6.1 cm (SDS -0.7±1). The adult height was within the target height range in all treated girls with CPP.

The correlations between the AH and PAH using the BP method with the advanced and average BA tables after the GnRHa treatment were the strongest (r=0.72 and r=0.74; *p*<0.001, respectively) (Table 3 and Figure 2).

### Prediction of age at menarche

The initial mean predicted age at menarche in the girls with ICPP using the formulae was 10.1±0.45 yr. After the GnRHa treatment, the mean age at menarche was significantly higher at 11.9±0.7 yr (*p*<0.001). The interval between the interruption of GnRHa treatment and occurrence of menarche was 1±0.6 yr.

## DISCUSSION

Among the main objectives of CPP treatment is to preserve the genetic adult height potential. The decision to treat CPP with GnRHa is based on several criteria, including an unfavorable PAH at the time of diagnosis 1-3,5-7. The Bayley-Pinneau method, which is the most commonly used method in clinical practice, relies on tables consisting of variables, such as BA and the patient's height at the clinical evaluation; this method has been an inaccurate tool 4,9,12,13. In the present study, we compared the PAH using the BP method with the average or advanced BA tables at the time of CPP diagnosis. The AH was overestimated using the BP method with the advanced BA tables, which is consistent with in previous studies 4,8. In addition, there are several limitations in establishing an accurate BA using the Greulich and Pyle method 10,11, which does not provide short intervals between ages. Therefore, other instruments using different variables for AH prediction should be developed.

In this study, to estimate the accuracy of the prediction methods, we retrospectively evaluated height predictions at the onset and end of puberty suppression in female patients with ICPP who reached their final height. The BP method was compared with formulae based on a mathematical model described and validated by Giabicani et al. 9. We demonstrated that the PAH using the BP tables for average and advanced BA at the time of CPP diagnosis was significantly shorter than the PAH after the GnRHa treatment, demonstrating the beneficial effect of pubertal suppression on the AH. Moreover, at the initial diagnosis, the AH predictions based on the Bayley-Pinneau tables underestimated the achieved AH in the treated CPP girls. In contrast, the AH was overestimated by the formulae from the mathematical model, even in girls who were not treated. Finally, after the GnRHa treatment, the PAH using the BP method with the average BA tables was the closest to the AH of the treated girls.

Based on the mean BA after the treatment (Table 2), we suggest that the prediction of the AH using the BP tables was more accurate with BA higher than 12.5 yr in girls. Here, all correlations between the BP method and the mathematical model for adult height predictions in patients with ICPP were weak or moderate, indicating that these methods are limited and must be used with caution. Notably, BA is observer-dependent, and hormonal levels are assay method-dependent, which could contribute to the high variability in the results.

Although Giabicani et al. 9 stated that GnRHa treatment for CPP has no or slight effects on AH, in our study, this treatment resulted in the preservation of the genetic height potential. However, a limitation of our study was the lack of a group composed of untreated girls.

Giabicani et al. 9 also developed formulae for predicting the age at spontaneous menarche if CPP is not treated. Although our population consisted of only treated girls, we provide evidence that GnRHa treatment significantly delays the predicted age of menarche.

In conclusion, the BP and mathematical methods for adult height prediction are limited. The best prediction was achieved using BP tables after GnRHa treatment with a BA higher than 12.5 yr in girls with CPP. The prediction of the age at menarche may serve as an innovative tool that is helpful for clinical decision-making.

## AUTHOR CONTRIBUTIONS

Lopes MC contributed to the acquisition and analysis of the data and wrote the manuscript. Ramos CO contributed to the acquisition of the data. Mendonça BB, Latronico AC, and Brito VN supervised the clinical study, followed the patients and performed a critical review. All authors were involved in writing the manuscript and have approved its final version.

## Figures and Tables

**Table 1:** Criteria used to diagnose idiopathic precocious puberty in girls and definition of adult height 1,2.

Criteria A – Phenotype of Precocious Puberty	Criteria B – Central Precocious Puberty (CPP)	Criteria C – Definition of Idiopathic CPP	Criteria D – Definition of Adult Height
✓ Tanner breast stage ≥ B2 in girls younger than 8 yr ✓ Bone age greater than 2 yr from the chronological age as established by hand X-ray ✓ Growth rate greater than +2 SD in the year prior to the first evaluation	✓ Basal LH>0.6 U/L (IFMA) or >0.2 U/L (ECLIA) OR ✓ LH Peak after GnRH test >6.9 U/L (IFMA) or >5 U/L (ECLIA)	✓ Normal central nervous system MRI ✓ No history of chronic exposure to sexual hormones ✓ No history of previous surgery for adrenal or gonadal tumors	✓ Bone age>16 yr AND/OR ✓ Growth rate less than 1 cm during the previous year

B: breast development.

**Table 2:** Clinical and hormonal data of 48 girls with ICPP.

Condition	Parameter	Mean	SDS
	CA at puberty onset (yr)	5.75	2.08
Initial evaluation	CA (yr)	7.75	1.7
	Interval to diagnosis (yr)	2.00	1.7
	BA (yr)	10.5	1.4
	Height (cm)	134.25	8.9
	Target height (cm)	157.8	6.1
	Target height (SDS)	-0.7	1.0
Hormonal profile	Basal LH (U/L)	1.57	1.33
	Basal FSH (U/L)	3.99	1.85
	Estradiol (pg/mL)	27.12	17.85
	LH peak (U/L)	17.54	15.56
	FSH peak (U/L)	13.24	7.07
	LHP/FSHP	1.33	0.79
Height prediction methods	BP for average BA (cm)	152.77	7.90
	BP for advanced BA (cm)	156.51	8.58
	Formulae from MM (cm)	160.04	4.95
Treatment	Duration (yr)	3.02	1.31
	CA (yr) at onset of treatment	7.89	1.25
After treatment	CA (yr)	10.91	0.73
	BA (yr)	12.50	0.68
	Height (cm)	149.22	5.05
Height prediction	BP for advanced BA (cm)	160.02	7.15
	BP for average BA (cm)	158.78	6.76
Menarche	CA (yr) at menarche	11.91	0.72
	Prediction of age at menarche (yr)	10.14	0.45
Adulthood	Height (cm)	158.40	6.20
	Height (SDS)	-0.63	1.0

CA: chronological age; BA: bone age; SDS: standard deviation score; BP: Bayley-Pinneau; MM: mathematical model.

**Table 3:** Correlations between distinct methods for AH prediction at diagnosis and after GnRHa treatment.

	Initial BP for Advanced BA (BPA1)	Initial BP for Average BA (BPN1)	Formulae from MM	Final BP for Advanced BA (BPA2)	Final BP for Average BA (BPN2)	AH
BPA1	NA	0.98; *p*<0.001	0.325; *p*<0.05	0.5; *p*<0.001	0.5; *p*<0.0001	0.45; *p*<0.001
BPN1	0.98; *p*<0.001	NA	0.4; *p*<0.01	0.52; *p*<0.001	0.5; *p*<0.001	0.48; *p*<0.001
MM	0.325; *p*<0.05	0.4; *p*<0.01	NA	0.36; *p*<0.01	0.4; *p*<0.001	0.58; *p*<0.001
BPA2	0.5; *p*<0.001	0.52; *p*<0.001	0.36; *p*<0.01	NA	0.99; *p*<0.001	0.72; *p*<0.001
BPN2	0.5; *p*<0.0001	0.5; *p*<0.001	0.4; *p*<0.001	0.99; *p*<0.001	NA	0.74; *p*<0.001
AH	0.45; *p*<0.001	0.48; *p*<0.001	0.58; *p*<0.001	0.72; *p*<0.001	0.74; *p*<0.0001	NA

BBPA: Bayley-Pinneau for advanced BA table; BPN: Bayley-Pinneau for average BA table; AH: adult height; MM: mathematical model; NA: not applicable.
